# Distribution of Cortical Endoplasmic Reticulum Determines Positioning of Endocytic Events in Yeast Plasma Membrane

**DOI:** 10.1371/journal.pone.0035132

**Published:** 2012-04-09

**Authors:** Vendula Stradalova, Michaela Blazikova, Guido Grossmann, Miroslava Opekarová, Widmar Tanner, Jan Malinsky

**Affiliations:** 1 Institute of Experimental Medicine, Academy of Sciences of the Czech Republic, Prague, Czech Republic; 2 Faculty of Mathematics and Physics, Charles University in Prague, Prague, Czech Republic; 3 Department for Plant Biology, Carnegie Institution for Science, Stanford, California, United States of America; 4 Institute of Microbiology, Academy of Sciences of the Czech Republic, Prague, Czech Republic; 5 Institute of Cell Biology and Plant Physiology, University of Regensburg, Regensburg, Germany; Université de Nice-CNRS, France

## Abstract

In many eukaryotes, a significant part of the plasma membrane is closely associated with the dynamic meshwork of cortical endoplasmic reticulum (cortical ER). We mapped temporal variations in the local coverage of the yeast plasma membrane with cortical ER pattern and identified micron-sized plasma membrane domains clearly different in cortical ER persistence. We show that clathrin-mediated endocytosis is initiated outside the cortical ER-covered plasma membrane zones. These cortical ER-covered zones are highly dynamic but do not overlap with the immobile and also endocytosis-inactive membrane compartment of Can1 (MCC) and the subjacent eisosomes. The eisosomal component Pil1 is shown to regulate the distribution of cortical ER and thus the accessibility of the plasma membrane for endocytosis.

## Introduction

Besides its basic function as a selective diffusion barrier, the plasma membrane (PM) hosts a variety of cellular functions including nutrient sensing and transport, sensing of various types of stress, endo- and exocytosis and signaling, and mediates the communication of the cell with its environment. To coordinate these processes and to ensure constant material and information exchange, the plasma membrane has to be precisely organized. Independent lines of evidence show that the membrane is compartmentalized into domains of specific structure and function [1].

Two stable membrane compartments were described in the plasma membrane of the yeast *S. cerevisiae* [2], [3]. The Membrane Compartment of arginine permease Can1 (MCC), corresponding to furrow-like plasma membrane invaginations [4], is organized by a cytosolic complex called eisosome [5]. Several possible biological functions of this specialized membrane compartment have been suggested to date, including a role in sphingolipid sensing and signaling [6] and regulation of protein turnover [7], the latter still being a matter of scientific debate [8]. The originally proposed involvement of eisosome in canonical endocytosis [5] has been ruled out [7], [8]. The PM area surrounding MCC was named MCP, referring to its first identified constituent, the major H^+^/ATPase Pma1 [2]. Dynamic processes apparently take place outside the highly stable MCC domains: endocytic and exocytic sites, for example, do not overlap with MCC [7], [8], and the formation of TORC2 signaling complexes occurs in the PM areas that contain neither MCC markers nor the MCP marker Pma1 [9]. The temporal order, in which the specific factors bind the sites of canonical endocytosis, has been described [10], [11], [12]. The above conclusion concerning the distribution of endocytic events in respect to MCC [7] was based on localization of Ede1, one of the first coat proteins arriving at the endocytic spot [13], [14], and thus reflected the process of endocytic site selection at the PM surface. However, the mechanism by which the sites of endo- and exocytosis are selected remains unclear.

In fungi, plants and also animals, a significant part of the cytosolic side of the plasma membrane is associated with the cortical endoplasmic reticulum (cortical ER) [15], [16], [17]. The cortical ER forms a dynamic meshwork in the close vicinity underneath the PM; sheets and tubules of the endoplasmic reticulum (ER) are continuously rearranged [16], [18], [19], [20]. Apart from its role in the secretory pathway, the ER establishes numerous membrane contact sites (MCS), connecting it with other membranous organelles in a cell, including the plasma membrane. In yeast, the best characterized MCS so far are nucleus-vacuole junctions [21], [22], whereas less is known about the composition and function of the others, including ER-PM contact sites. The upper distance limit defining ER-PM contact sites in yeast was set by Pichler and coworkers at about 30 nm. More than a thousand ER-PM MCS per yeast cell were identified by this set-up [19]. Only recently, the ER-PM spacing was measured more exactly to vary from 16 to 59 nm with a mean value of about 33 nm [20]. This indicates that the majority of the PM-associated cortical ER may be at a distance suitable for MCS formation.

Here we address the question whether close association of cortical ER and PM could locally affect endo- and exocytosis. The formation of an endocytic vesicle about 50 nm in diameter [23] may require accessibility to cytosolic components. For a substantial part of the PM inner surface [20], the cortical ER could represent a spatial hindrance for vesicular formation and/or delivery. It is known that cytoplasmic factors can modulate the PM organization. In yeast, actin filaments, for example [24], deliver various cargoes to the plasma membrane, and eisosomes, sub-membrane protein clusters organize protein and lipid distribution therein [25], [26], [27]. In the case of cortical ER, however, it would be the shape of a membranous cytoplasmic organelle that influences the local functional properties of the plasma membrane. We show that the endocytic machinery is positioned and functional only at PM sites free of cortical ER. The cortical ER pattern, on the other hand, is influenced by the association of eisosomes with MCC.

## Results

### Distribution of cortical ER with respect to endocytosis and MCC

**Figure 1:**
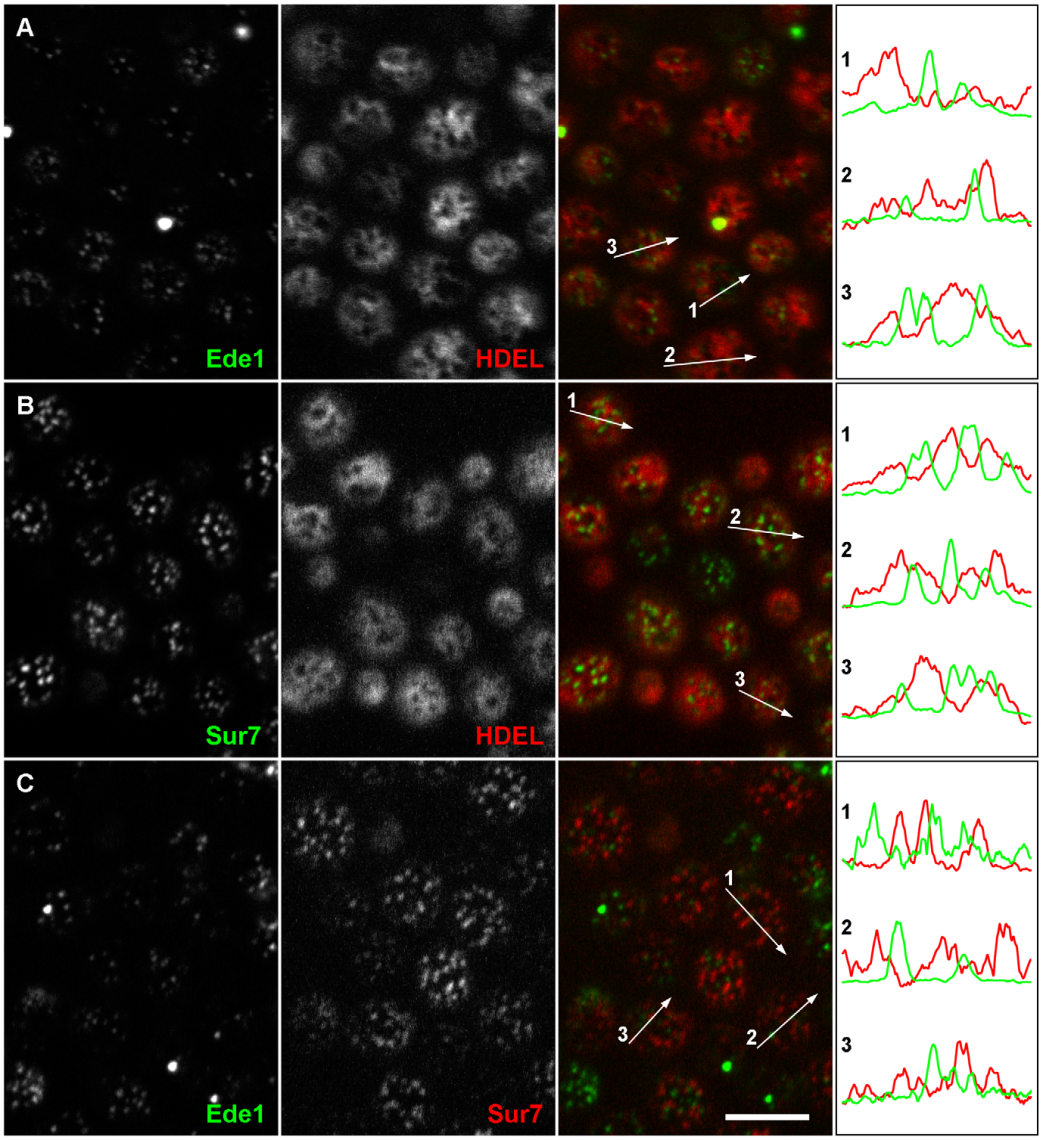
Endocytosis is initiated in the ER-free zones of the plasma membrane. Mutual localization was performed for Ede1-GFP, a marker of early stages of endocytosis, and cortical ER visualized by ss-dsRed-HDEL. Only rare colocalization events were detected (**A**). Similarly, the cortical ER network (**B**) and the initiation sites of endocytosis (**C**) were not colocalized with MCC domains marked with Sur7-GFP and Sur7-mRFP, respectively. Tangential confocal sections of BY4741 cells expressing fluorescently labeled proteins are presented. The fluorescence intensity profiles along the numbered arrows were scaled to the same range in the red and green channels. Bar: 5 µm.

To test whether endocytosis occurs at sites equally distributed throughout MCP of the yeast plasma membrane or whether the close apposition of the cortical ER network to the PM results in a non-random appearance of endocytic events, we monitored endocytic events with respect to the presence of cortical ER in the cortex of isotropically growing mother cells. We chose Ede1-GFP as an endocytic marker because it is one of the first proteins arriving at the endocytic spot [13], [14], and the ER luminal marker ss-dsRed-HDEL [28]. By observation of exponentially growing living yeast cells expressing both the markers we were able to visualize endocytic events and cortical ER simultaneously. As is evident from tangential confocal sections, the initiation of endocytosis occurred almost exclusively in PM zones not occupied by cortical ER (Fig. 1A). As a control, we included MCC into this mutual localization analysis and colocalized mCherry- and GFP-tagged versions of the MCC constituent Sur7 [29] with the above markers for ER and endocytic sites. In agreement with previous findings [4], [7], [8], we observed MCC domains not colocalized with either of these markers (Fig. 1B, C). Quantification of the entire dataset of acquired images revealed that markers of all three studied cortical structures (MCC, endocytic sites, and cortical ER) occupied three separate domains in the PM (Table 1). This PM partitioning seems to be independent on the yeast strain background as BY4741 and W303-1A cells yielded identical results (Table 1, compare also Fig. 1 and Fig. S1).

**Table 1:** Quantification of mutual localization of MCC, Ede1 sites and cortical ER.

Analyzed structures	BY4741	W303-1A
Ede1/cortical ER	93±10% (n = 144 cells)*	94±8% (n = 148)*
Sur7/cortical ER	98±3% (n = 139)**	98±4% (n = 142)**
Ede1/Sur7	99±4% (n = 150)*	99±5% (n = 160)*

* fraction of Ede1 sites non-colocalizing with cortical ER or the Sur7 signal.

** fraction of Sur7 domains non-colocalizing with the cortical ER signal.

**Figure 2:**
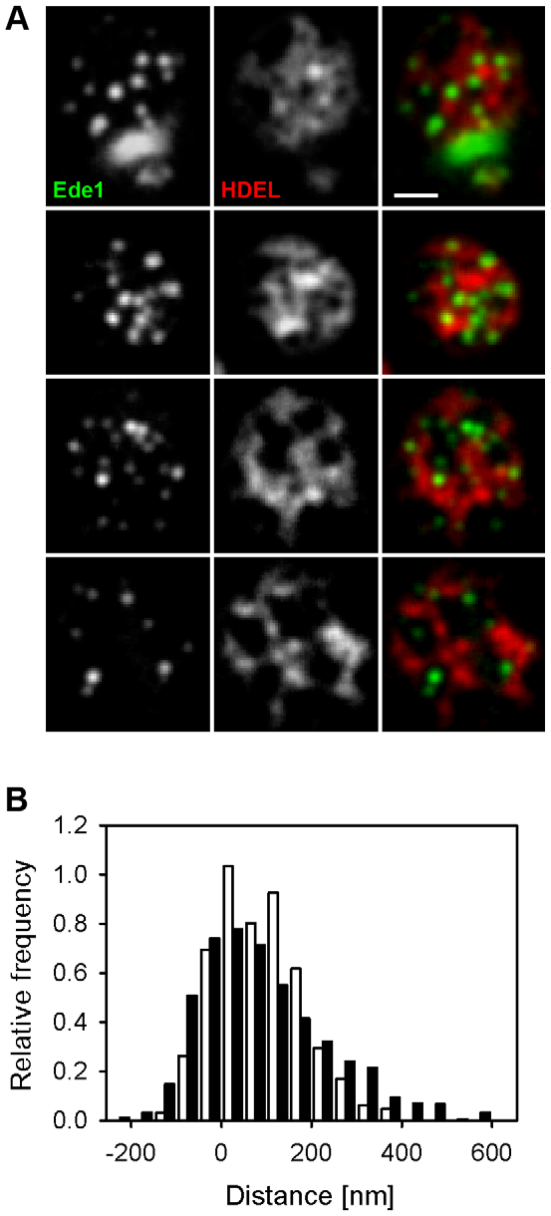
Endocytic events are randomly distributed within ER-free PM areas. In tangential confocal sections of individual W303 cells expressing Ede1-GFP and ss-dsRed-HDEL (**A**), the minimal distance of endocytic sites from the cortical ER boundary was measured. The histogram of the measured distances (full bars in **B**; 906 sites in 200 cells were analyzed) was compared to the distribution of the distances of model foci randomly positioned in the plasma membrane (empty bars in B; 320 foci in 100 cells; see Methods for details). In order to maximize the accuracy of the distance measurements, for all the measurements we chose only the foci located to easily discernible ER holes positioned in central parts of the tangential confocal sections, so that the entire borders of the holes could be traced. Bar: 1 µm.

Then we attempted to describe the distribution of endocytic events in more detail. We measured the minimal distance of endocytic spots in areas not covered by the cortical ER (holes) to the ER network. We defined the ER boundary as a line connecting points that exhibited half of the local intensity drop between the signal of the ER marker ss-dsRed-HDEL and the hole. Adaptive character of this border definition makes it independent on signal intensity and thus more reliable than any threshold-based definition. The method could lead to an overestimation of the real ER size in the range of ∼100 nm in any direction, since defining the ER border in this way possibly includes the blur of the fluorescence signal of the ER marker. We measured the distance between this cortical ER border and maxima of the Ede1 signal. As a control, we generated a set of images containing foci randomly distributed in the plasma membrane over the cortical ER pattern (see Methods for details). We selected about 30% of these foci, which localized into the holes in the ER pattern, and again measured their distance from cortical ER. Comparison of the two distributions revealed that endocytic events are randomly positioned within the free-of-ER plasma membrane, with a weak preference of places lying further apart from cortical ER border (Fig. 2, note the asymmetry of the endocytic foci distribution). Nonetheless, this means that most of the endocytic sites are selected at the plasma membrane not further than 200 nm from the ER border (Fig. 2). Similar analysis of MCC foci distribution revealed that MCC is also randomly distributed in areas devoid of cortical ER coverage. But, in contrast to Ede1 and random foci, the fluorescence signal of Sur7 remains some minimal distance from the ER border (see the symmetric distribution of Sur7 foci in Fig. S2).

### Local variations in spatio-temporal distribution of cortical ER

**Figure 3:**
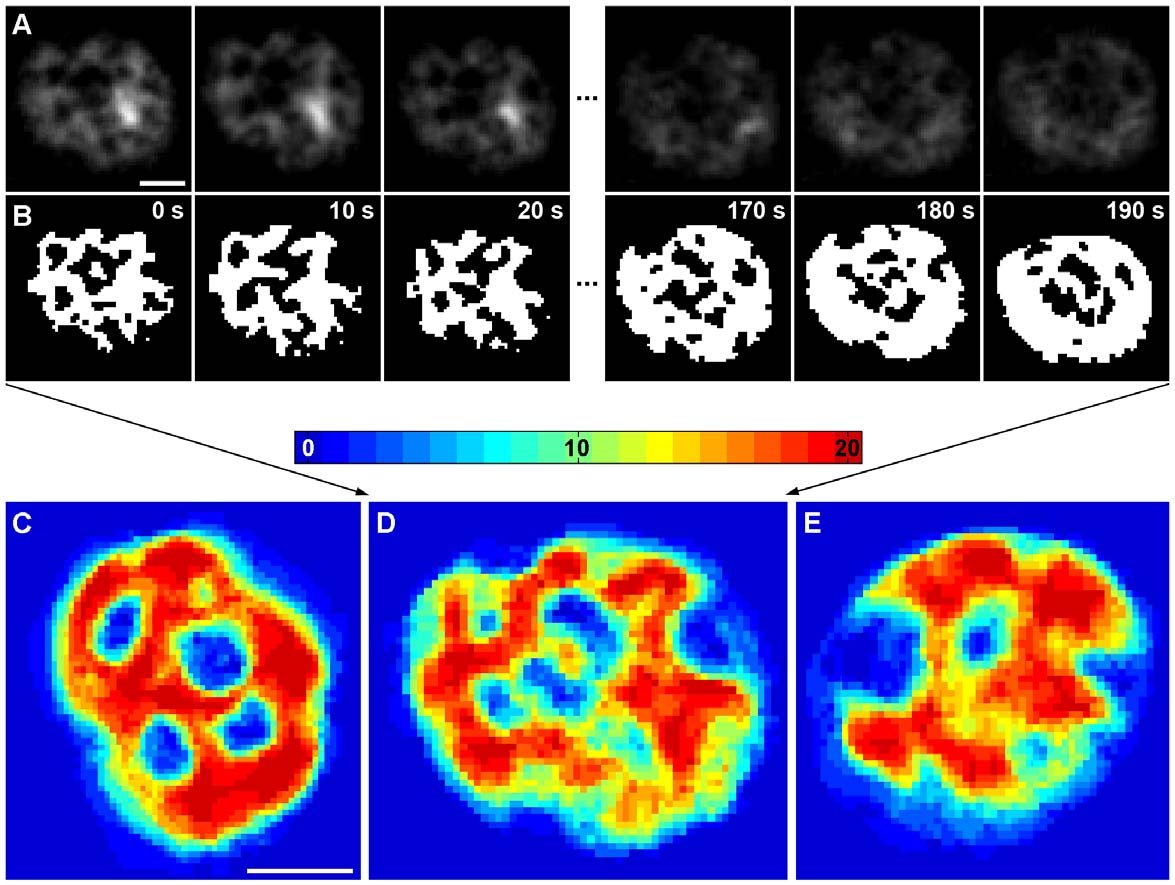
Differential cortical ER coverage defines micron-scale PM domains. The dynamics of cortical ER was followed in time-lapse series of 20 tangential confocal sections of BY4741 cells expressing ss-GFP-HDEL together with Sur7-mCherry (rate: 10 s/frame). Raw data after a 3×3 mean filtration (**A**) and binarized cortical ER pattern (**B**) of the first and the last three frames in the series are presented. For better lucidity, the red fluorescence channel (MCC/Sur7-mCherry) is not shown. In order to visualize the local dynamics of cortical ER, all twenty binarized frames were superimposed. Three out of 33 cells analyzed are presented in false colors denoting the number of frames in the series in which cortical ER was detected (**C–E**). Bar: 1 µm.

The network of cortical endoplasmic reticulum represents a highly dynamic organelle undergoing continuous rearrangement that may further contribute to the dynamic accessibility of the plasma membrane for membrane trafficking. To track the dynamics of cortical ER, we measured the movement of GFP-HDEL stained ER (Fig. 3A) in Sur7-mCherry expressing cells. The immobile, Sur7-mCherry-labeled MCC domains were used for alignment of 20 consecutive frames (increment: 10 s/frame) in a time-lapse series. Consistent with earlier observations reporting 57–77% of the cell periphery to be covered by cortical ER [18], [19], [30], we observed a GFP-HDEL signal over 65±8% of the PM surface (n = 30 series; 20 frames each). The binarized cortical ER patterns (Fig. 3B) were superimposed in order to visualize the local durations of plasma membrane coverage with cortical ER during the monitored time window. Within 3 minutes, almost the entire area of PM (98.7±1.3%) was covered at least once by cortical ER. 9.3±3.8% of the PM surface was covered with cortical ER permanently (dark red areas in Figs. 3C–E). This visualization allowed for identification of micron-scale PM zones with strikingly diverse relative cortical ER coverage. While domains that are almost permanently covered by cortical ER exist on the inner surface of PM (red and orange zones in Fig. 3), other zones barely came in contact with cortical ER (blue in Fig. 3).

**Figure 4:**
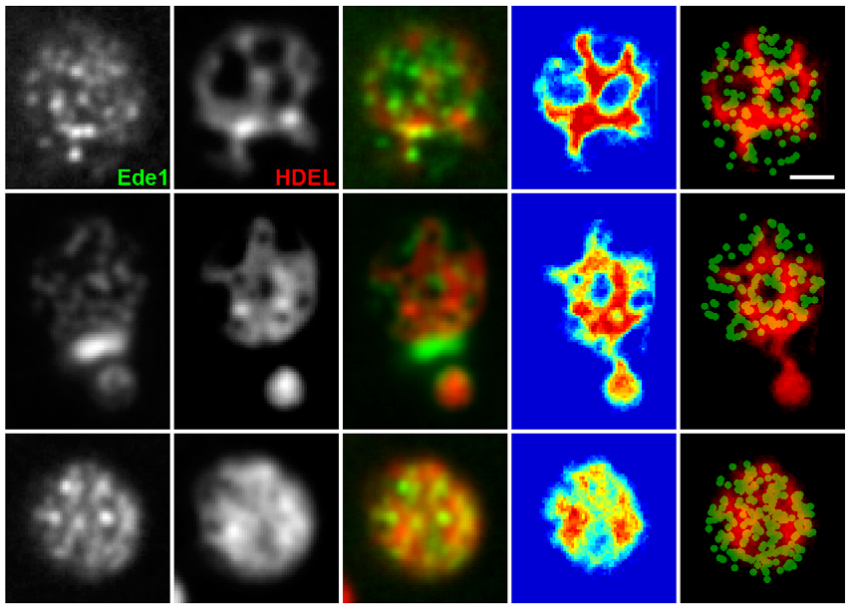
Lateral mobility of cortical ER determines the positioning of endocytic events. Initiation of endocytic events in cells co-expressing Ede1-GFP and ss-dsRed-HDEL was monitored in a time-lapse experiment (20 frames, 30 s/frame). Superposition of all the frames is presented. The 4^th^ column represents the superimposed binarized ER signals from 20 consecutive frames in a false-color blue-to-red scale to highlight the dynamics of the cortical ER network (see Fig. 3 legend for an explanation). The column on the far right shows this superposition of binarized ER signals in red overlaid by the green channel, in which the positions of the maxima of the Ede1 sites in the 20 frames were marked by round spots. Bar: 1 µm.

We propose that the cortical ER is involved in functional compartmentalization of the PM as it confines the immediate communication between the PM and cytosol to distinct (ER-free) zones. This becomes clearly visible when the Ede1-GFP signal is accumulated in time. In cells exhibiting low cortical ER dynamics, Ede1-GFP appears in isolated domains within the plasma membrane surrounded by ER. During the same time period, cells with higher ER dynamics become evenly covered with Ede1-marked sites (Fig. 4).

The only stable parts of the PM in our study are the MCC areas, which are not accessible either for interaction with ER or for initiation of endocytosis. Our analysis showed that both cortical ER and endocytic sites can extend to the rest of the plasma membrane, although their immediate plasma membrane distributions at any given time do not overlap (Figs. 3, 4).

### Pil1 influences the cortical ER network spreading

Previously, we have shown that the cortical ER does not overlap with the stable, invaginated MCC/eisosome domains (Fig. 1B and Fig. S2A) [4]. The invaginations stretch into the cytosol with a depth of about 50 nm and may possibly cause a hindrance for lateral cortical ER spreading. We tested whether mutants being affected in MCC/eisosome distribution or invagination show an altered cortical ER arrangement beneath the PM.

**Figure 5:**
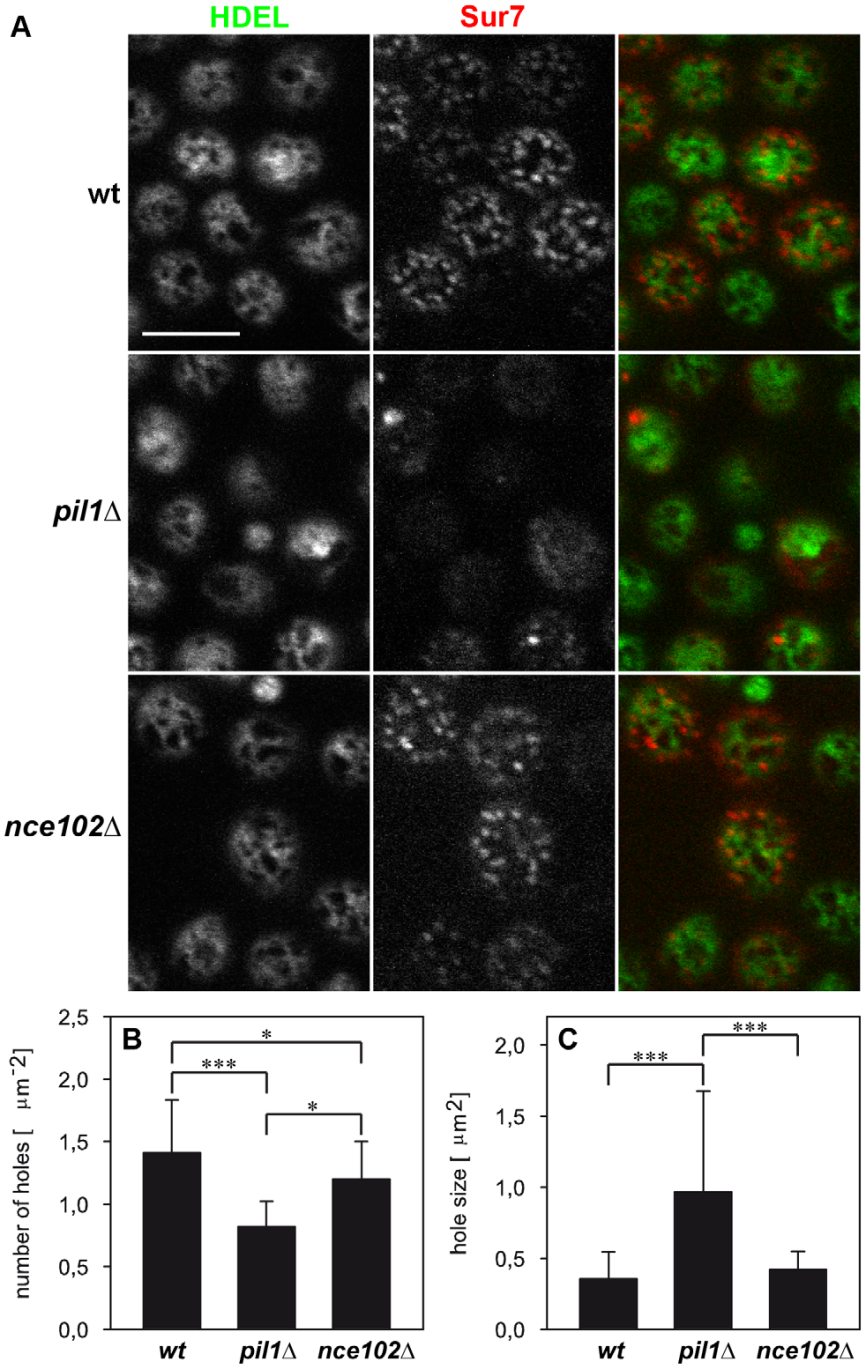
Defect in MCC integrity results in alteration of the cortical ER pattern. Tangential confocal sections of BY4741, *pil1Δ* and *nce102Δ* cells expressing ss-GFP-HDEL and Sur7-mCherry markers are presented (**A**). Statistical analysis of cortical ER pattern in all strains (n>30) revealed that the cortical ER network in *pil1Δ* cells exhibits fewer (**B**) but larger (**C**) holes. Importantly, no difference in total cortical ER area with respect to the individual tested strains was detected. Mean values (± standard deviation) are compared and the significance of detected effects as revealed by Student T test is denoted (* p<0.05;***p<0.001). Bar: 5 µm.

**Figure 6:**
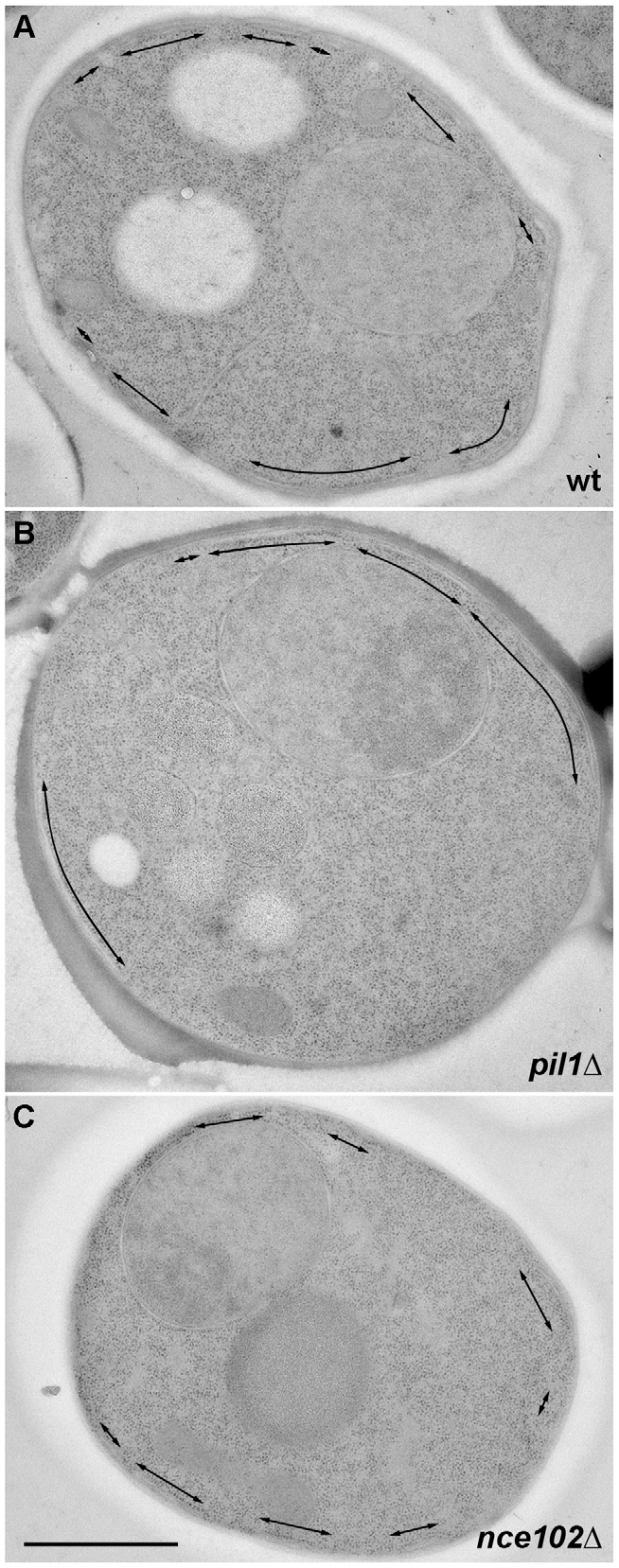
Electron microscopic analysis of the MCC-specific alterations of cortical ER pattern. The length and distribution of cortical ER cisternae (arrows) on thin sections of BY4741 (**A**), *pil1Δ* (**B**) and *nce102Δ* cells (**C**) were compared. No difference in total length of the cortical ER structures with respect to the individual tested strains was detected. Bar: 1 µm.

First, we explored the cortical ER pattern in strains *pil1Δ* [5] and *nce102Δ* [7] which were shown to be defective in MCC appearance. The fluorescence pattern of cortical ER resembles a network: the compact labeled areas appear fragmented by circular or irregularly shaped holes (perforations) into a system of more or less fibrous (tubular) structures and sheets (cisternae). We detected morphological changes of this network in selected mutants. In *pil1Δ* cells, in which both MCC and the eisosome structure are disrupted [5], the cortical ER network was generally more compact and indiscrete (Fig. 5A). Quantitative analyses revealed a cortical ER with fewer but larger perforations as compared to the wild type (Fig. 5B, C, Fig. S3 and Movies S1,S2,S3). Accordingly, less fragmented, unequally distributed tubuli and cisternae of cortical ER were also clearly discernible beneath the plasma membrane of ultrathin-sectioned *pil1Δ* cells (Fig. 6) observed by the transmission electron microscope. On the other hand, cells over-expressing Pil1, which were reported to contain more eisosomes than the wild type [31], exhibited cortical ER with a high number of smaller holes (Fig. S4). In *nce102Δ* mutant cells eisosomes correctly localize beneath MCC domains [6], [32], but membrane invaginations are lacking [4]. Testing the distribution of cortical ER in *nce102Δ* cells did not reveal any significant alteration in the cortical ER morphology (Figs. 5, 6), indicating that the invagination of MCC domains is not required to restrain the cortical ER from spreading over MCC areas.

**Figure 7:**
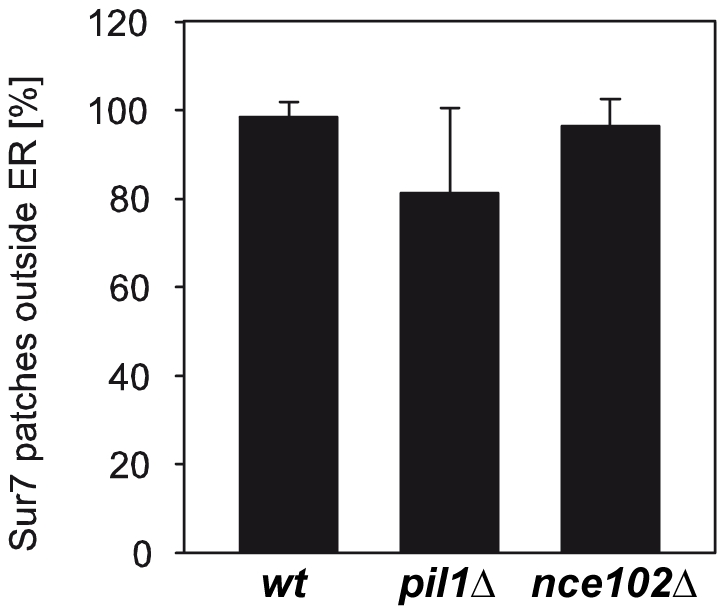
Distinct localization of MCC marker Sur7 and cortical ER distributions is maintained in MCC-defective strains. The mutual position of fluorescence signals in tangential confocal sections of BY4741, *pil1Δ* and *nce102Δ* cells co-expressing Sur7-GFP and ss-dsRed-HDEL (n>140) was analyzed. Relative numbers (mean ± standard deviation) of Sur7-GFP domains localized outside the ss-dsRed-HDEL (cortical ER) pattern are compared.

To test whether the distinct localization of MCC and cortical ER is preserved in the two MCC defective strains, we analyzed the localization of ss-dsRed-HDEL and Sur7-GFP in *pil1Δ* and *nce102Δ* cells. In agreement with previously published data [6], we found a lower surface density of Sur7 domains in *nce102Δ* cells (0.75±0.19 µm^−2^) as compared to the wild type (1.21±0.27 µm^−2^). In *pil1Δ* cells, the Sur7 protein was originally reported to be clustered only into occasional big “eisosome remnants” and otherwise homogenously distributed in the membrane [5]. In agreement with our earlier observations [7], we show on tangential confocal sections of *pil1Δ* cells that aside the eisosome remnants Sur7-GFP was also not completely evenly distributed, but rather concentrated into smaller, less distinct domains (Fig. S5). We analyzed the percentage of overlap between Sur7 domains and cortical ER in *nce102Δ*and *pil1Δ* cells, including all discernible Sur7 domains in *pil1Δ*, and found no significant difference between the tested strains (Fig. 7). Thus we demonstrate that, even under the conditions when Sur7 is not concentrated in large, easily distinguishable domains, the protein localizes preferentially to PM areas devoid of cortical ER coverage. We also examined and quantified the positioning of endocytic Ede1-GFP sites in respect to the cortical ER area in the two mutant strains. Again, endocytosis occurred solely outside the ER-covered PM areas, as about 94% of endocytic sites did not colocalize with cortical ER network in either of *nce102Δ* or *pil1Δ* cells (data not shown).

Finally, to check whether the dynamics of ER rearrangement is affected upon MCC disintegration, we explored the rate of ER network movement in GFP-HDEL/Sur7-mCherry expressing strains. The Sur7-mCherry domains were again used to align the frames in the time-lapse series. We used a relative displacement, i.e. proportion of the area covered by cortical ER at a given time but not covered after a selected time interval, as a measure of the ER mobility. Mainly due to cell-to-cell variations in ER mobility, no significant difference in the dynamics of cortical ER rearrangements between the wild type and *nce102Δ* or *pil1Δ* cells was detected (Fig. S6A). Similar to WT, we were also able to detect the micron-scale plasma membrane zones exhibiting different cortical ER coverage in both the mutant strains. Consistent with the above-mentioned observation of altered cortical ER distribution in *pil1Δ* cells (Fig. 5 and Fig. S3), the pattern of these domains was changed accordingly (Fig. S6B; compare with Fig. 3).

We conclude that the eisosome core protein Pil1 influences the distribution of the cortical ER network. Pil1 is not required for normal cortical ER mobility or PM binding but Pil1-driven eisosome formation rather contributes to cortical ER fragmentation.

## Discussion

Directional targeting of material exchange-related processes like endo- and exocytosis is one of the basic processes enabling a living cell to exist. For clathrin-mediated endocytosis, a detailed picture of the order and timing has been acquired by Drubin and coworkers [11]. The regulation and spatial distribution of sites of endocytosis, however, remain elusive. In our study, we used baker's yeast for mapping endocytic events in relation to cortical ER and the known, immobile domains of MCC, which are regulated by the subjacent eisosomes. We found sites of endocytosis to be non-randomly distributed and restricted to sites free of cortical ER and free of MCC. Cortical ER distribution itself also appears to be dependent on normal MCC-eisosome formation.

Specific re-positioning of the ER during the process of budding was described in detail in a recent study [20]. We report that apart from this polar organization of the cellular cortex, the uneven and variable distribution of cortical ER can be followed within the plasma membrane of *S. cerevisiae*. We found micrometer-sized domains preferentially covered by the cortical ER network and domains preferentially free of this coverage coexisting within the PM in a time scale of minutes (Fig. 3).

As evident from the Ede1-cortical ER co-labeling experiments (Fig. 1), plasma membrane areas devoid of cortical ER coverage determine the emergence of endocytic vesicles. Being a soluble, cytosolic protein, Ede1 is one of the first proteins marking the future site of endocytosis by clustering at the plasma membrane [13], [14]. Even if its movement in cytoplasm was driven solely by diffusion, it is likely that its interaction with PM would occur preferentially in the membrane domains that are not covered with cortical ER. Our quantifications show that this preference is very strong (93±10%). Recently, a similar mechanism of indirect soluble protein routing in the cell cortex has been observed in *S. pombe*: the actomyosin ring organizing protein Mid1 is directed to a cortical ER-determined “permissive zone” in the plasma membrane, in which the plane of cell division (cytokinesis) is consequentially established [33]. The distribution of Ede1 foci in plasma membrane domains not covered with cortical ER is rather random (Fig. 2), thus indicating that initiation of endocytosis is independent from the lateral distance to cortical ER as long as the plasma membrane is not covered with ER.

Plasma membrane invaginations (MCC domains) and eisosomes seem to modulate the ratio of cortical ER tubules to extended cisternae. Contrary to our expectations, we found that the depth of furrow-like PM invaginations/eisosome has no, or only a minor influence on the cortical ER morphology. Rather the presence or absence of MCC/eisosomes, independent of the local PM topology, determines the local perforation of the cortical ER network. Several lines of evidence allow for this conclusion. First, these two structures localize in distinct parts of the plasma membrane (Fig. 1, Fig. S1) as we also reported previously [4]. More significantly, a lack of the eisosomal Pil1p results in fewer MCC/eisosome domains and fewer holes in the pattern of cortical ER (Figs. 5, 6). Pil1 overexpression, however, increases the number of MCC/eisosome domains and perforations of cortical ER network (Fig. S4). Even in the absence of specific MCC-ER interactions, it can be difficult for the ER sheet or tubulus to enter between adjacent eisosomes, which are as frequent as 2.5±0.2 um^−2^ in the plasma membrane surface and about 300 nm long each (parameters reported by a freeze-etching study in Stradalova et al., 2009), simply because of mechanical obstacles. The surface tension in the ER membrane and the fact that the formation of curved areas in the ER membrane anticipates the assistance of specific lipids will contribute here. In any case, we do observe high ER dynamics beneath the plasma membrane. Thus, one can assume that these obstacles are not impossible to overcome and that ER still can enter most of inter-MCC gates visible at the resolution of fluorescence microscopy.

**Figure 8:**
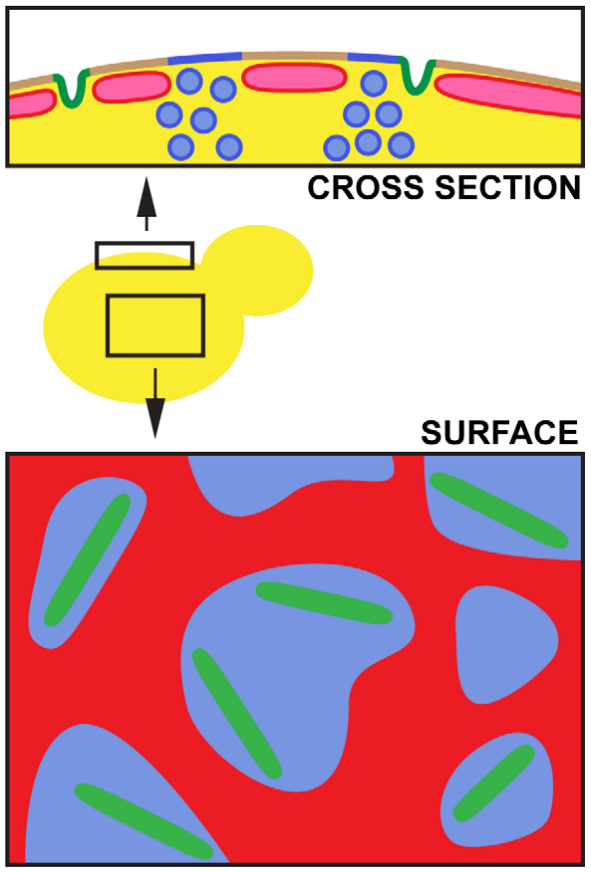
Model of functional plasma membrane compartmentalization in yeast. Three distinct domains can be distinguished in the yeast PM: stable MCC domains (green) surrounded by a membrane covered with a dynamic network of cortical ER (red) and the PM free of cortical ER coverage (blue). Vesicle transport can take place only in the ER-free PM.

In contrast to stable MCC/MCP partitioning of the plasma membrane, the dynamics of membrane areas with differential cortical ER coverage is much higher. We do not expect, therefore, that discriminative protein or lipid markers of these domains will be found in the PM. One can rather imagine that cortical ER distribution supports more or less stable gradients of the PM constituents as it directs the flows of cytoplasmic soluble factors and vesicles towards certain zones of the PM. Similar steady-state modulation of the PM structure/function was recently suggested in plants: polar accumulation of auxin transporter Pin2 was reported to result from spatially defined exo- and endocytosis in *Arabidopsis* [34]. Based on the present knowledge, we propose a simplified scheme of yeast cell cortex showing its spatial and consequent functional map (Fig. 8).

## Materials and Methods

### Yeast strains and growth conditions

The yeast strains used in this study are listed in Table S1. The cells were grown in a synthetic complete medium (0.67% Difco yeast nitrogen base without amino acids, 2% glucose, and amino acids; for W303-derived strains supplemented with 2× more adenine) to mid-log phase (OD_600_ about 0.6) at 30°C on a shaker. The cells in Figure S4 were cultivated in synthetic medium lacking uracil. For electron microscopy preparations, the cells were cultured in a rich medium (YPD; 2% peptone, 1% yeast extract, 2% glucose) to the same OD_600_ and under the same conditions as the cells in synthetic medium.

### Plasmids

**YIp211-Ede1-GFP** [**7**] **; YIp211-Sur7-GFP** [**35**] **; YIp128-Sur7-mRFP**: The SUR7 gene was inserted as a HindIII-BamHI fragment into YIp128-mRFP plasmid [35]; **YIp211-Sur7-mCherry**: The mCherry gene was cut from pVTU100-HUP1-mCherry (G. Grossmann, unpublished) plasmid using BamHI-BssHII restriction sites and ligated into YIp211-SUR7-GFP plasmid instead of the GFP gene. Before transformation, the plasmid was linearized by digestion with EagI; **YIp204-TKC-dsRed-HDEL** [28]; **YIp128-TRP1-TKC-dsRed-HDEL**: The TRP1-TKC-dsRed-HDEL cassette was amplified by PCR from the YIp204-TKC-dsRed-HDEL plasmid using the primers HDEL_FW (GATTACGCCAAGCTTGCAAATTAAAGC) and HDEL_RV (CTTGGAGCTCGTCTGTTATTAATTTCAC). The obtained fragment was ligated into YIp128 plasmid using the HindIII-SacI restriction sites. The plasmid was linearized before yeast transformation by Bsu36I enzyme and integrated into TRP1 locus; **YIp128-TRP1-TKC-GFP-HDEL**: The GFP gene was amplified by PCR from the YIp211-SUR7-GFP plasmid using the primers VN155_HDEL_F (TATAGGATCCCATGTCTAAAGGTGAAG) and VC155_HDEL_R (TATATCTAGATTACAATTCGTCGTGTTTGTACAATTCATC). The obtained GFP-HDEL fragment was inserted into 128-TRP1-TKC-dsRed-HDEL via BamHI-XbaI restriction sites instead of dsRed-HDEL gene. The plasmid was linearized before yeast transformation by Bsu36I enzyme and integrated into TRP1 locus; **pVTU100-Pil1-mRFP**: The PIL1 gene was inserted as a HindIII-BamHI fragment into pVTU100-mRFP plasmid [32] under the ADH1 promoter.

### Confocal microscopy

Living yeast cells in synthetic medium were concentrated by brief centrifugation, immobilized on a 0.17 mm coverglass by a thin film of 1% agarose prepared in the synthetic complete medium and observed using LSM510-META confocal microscope (Zeiss) with a 100× PlanApochromat oil-immersion objective (NA = 1.4), with the exceptions listed below. Fluorescence signals of GFP and mRFP/dsRed/mCherry (excitation 488 nm/Ar laser, and 561 nm/solid state laser) were detected using the 505–550 nm band-pass, and 580 nm long-pass emission filters, respectively. Cells for Fig. 3 and Figs. S2, S6 were visualized using Zeiss/Yokogawa Cell Observer spinning disc microscope with a 100× PlanApochromat oil-immersion objective (NA = 1.4); cells for Fig. 2 and Fig. 4 were visualized using Zeiss/Yokogawa Axio Observer.Z1 spinning disc microscope with a 100× PlanApochromat oil-immersion objective (NA = 1.46). The fluorescence signals of GFP and mCherry were detected using band pass emission filters (520/35 and 617/73 nm, respectively) and recorded using a Andor iXon+ 888 back-illuminated EMCCD camera (Fig. 3, S2 and S6) or AxioCamMR3 camera (Fig. 2 and 4).

### Electron microscopy

Yeast cells were processed as described previously [4], in brief: cells were filtered, loaded in a flat specimen carrier and frozen in EM PACT (Leica). Frozen samples were freeze-substituted in acetone supplemented with 3% glutaraldehyde (10% stock in acetone; SPI Supplies, USA), 0.1% UA and 1% water in AFS machine (Leica) and then embedded in HM20 resin. Ultrathin sections (70 nm) were cut with Ultracut S ultramicrotome equipped with a diamond knife (35°; Diatome) and placed on copper formvar-coated grids. Sections were contrasted with a saturated aqueous solution of UA for 1 hour, washed, air-dried and examined in a FEI Morgagni 268(D) transmission electron microscope at 80 kV. Images were captured with MegaView G2 CCD camera (Olympus).

### Image processing and evaluation

If not stated otherwise, raw microscopic data are presented. Mutual localization of MCC, endocytic sites, and cortical ER was evaluated manually as follows: any overlap between the two fluorescence channels was considered as a colocalization event. The only exception from this rule was the case when focal accumulation of endocytic or MCC marker overlapped with a local minimum in the cortical ER pattern. This particular case was evaluated as non-colocalization (small hole in the ER pattern, partially filled with the blur from the surroundings). Processing of time-lapse image series (Figures 3 and S6) (alignment, [3×3] mean filtering, binarization) was performed in Matlab software (The MathWorks): the positions of the MCC domains were determined as local maxima of the Sur7-mCherry signal. A convex hull using the Delaunay triangulation was constructed to determine the cell shape. Combination of thresholding and morphological operations was used to determine the inner structure of the cortical ER – positions and shapes of the perforations in the cortical ER pattern. Images for Figs. 2, 4 and S2 were filtered as stated above; the alignment for Fig. 4 was treated manually.

In analysis of the distances between the Ede1-GFP (Sur7-mCherry) domains and the cortical ER, images with random population of foci (the control for surface distribution analysis) were obtained as follows: the ER channel in the real images previously analyzed was left untouched. The fluorescence signal in the focal (MCC) channel was replaced by a regular hexagonal lattice of Gaussian foci (frequency: 1.2 µm^−2^). Only the foci falling inside the convex hull of the ER fluorescence signal were taken into account.

## Supporting Information

Figure S1 **Endocytosis is initiated in the ER free zones of the plasma membrane in W303-1A cells.** Mutual localization of Ede1-GFP, a marker of early stages of endocytosis, and cortical ER visualized by ss-dsRed-HDEL was performed. Only rare colocalization events were detected (**A**). Similarly, cortical ER network and initiation sites of endocytosis were not colocalized with MCC domains marked with Sur7-GFP (**B**) and Sur7-mRFP (**C**), respectively. Tangential confocal sections of W303-1A cells expressing fluorescently labeled proteins are presented. Fluorescence intensity profiles along the numbered arrows were scaled to the same range in the red and green channels. Bar: 5 µm.
(TIF)

Figure S2 **Distribution of MCC domains through the holes in the cortical ER pattern.** In tangential confocal sections of individual cells expressing Sur7-mCherry and GFP-HDEL (**A**), the minimal distance of the Sur7 labeled MCC domains from the cortical ER boundary was measured. The histogram of the measured distances (full bars in **B**; 399 foci in 64 cells were analyzed) was compared to the distribution of the distances of model foci randomly positioned in the plasma membrane (empty bars in B; 320 foci in 100 cells; see Methods for details). The Gaussian fits of the distributions are also depicted (Sur7 solid, randomly positioned foci dotted). In order to maximize the accuracy of the distance measurements, for all the measurements we chose only the foci located to easily discernible ER holes positioned in central parts of the tangential confocal sections, so that the entire borders of the holes could be traced Bar: 1 µm.
(TIF)

Figure S3 **Cortical ER pattern in MCC-defective strains.** Transparency projections (LSM Image Browser) of ER patterns in BY4741, *pil1Δ* and *nce102Δ* cells expressing ss-GFP-HDEL and Sur7-mCherry markers are compared. Only the green (ER) fluorescence pattern is presented. More projections of the same cells see also in Movies S1,S2,S3. Bar: 5 µm.
(TIF)

Figure S4 **Overexpression of Pil1 leads to increased fragmentation of the cortical ER pattern.** Cells co- expressing ss-GFP-HDEL (green) and Pil1-mRFP (red) under a strong promoter (strain VSY177) were observed. Compare the number of MCC/eisosomes and the number of cortical ER holes with those of wild type (Fig. 1) and *pil1Δ* cells (Fig. 5). Superposition of two consecutive confocal sections is presented. Bar: 5 µm.
(TIF)

Figure S5 **Distribution of Sur7 in** ***pil1Δ*** **cells.** Tangential confocal sections of *pil1Δ* cells expressing ss-dsRed-HDEL and Sur7-GFP markers (only green fluorescence channel visible) are presented. Note that, in addition to large and intensive “eisosome remnants”, smaller and less intensive local accumulations of Sur7-GFP are discernible in the surrounding membrane. Bar: 5 µm.
(TIF)

Figure S6 **Speed of cortical ER movement is not affected in MCC defective strains.** The speed of the cortical ER movement was measured as a decrease in the mutual overlap of the ss-GFP-HDEL patterns detected in living BY4741 (white), *pil1Δ* (grey) and *nce102Δ* (black) cells (n>30) after an increasing interval of time (**A**). The dynamics of cortical ER was followed in a time-lapse series of 20 tangential confocal sections of *pil1Δ* cells expressing ss-GFP-HDEL together with Sur7-mCherry (rate: 10 s/frame). For better lucidity, the red fluorescence channel (MCC/Sur7-mCherry) is not shown. The data were processed and binarized as shown in Fig. 3 and all twenty binarized frames were superimposed to visualize the local dynamics of cortical ER. Three out of 30 cells analyzed are presented in false colors denoting the number of frames in the series in which cortical ER was detected (**B**). Bar: 1 µm.
(TIF)

Table S1 **Strains used in this study.**
(DOC)

Movie S1 **Maximum intensity projections of ER pattern in BY4741cells.** Twenty-one MIP (Maximum Intensity Projections) of BY4741 cells from Fig. S1 in 3° angle increment (−30 to +30°) were calculated and joined. Presentation speed: 10 frames/s.
(AVI)

Movie S2 **Maximum intensity projections of ER pattern in** ***pil1Δ*** **cells.** Twenty-one MIP of *pil1Δ* cells from Fig. S1 in 3° angle increment (−30 to +30°) were calculated and joined. Presentation speed: 10 frames/s.
(AVI)

Movie S3 **Maximum intensity projections of ER pattern in** ***nce102Δ*** **cells.** Twenty-one MIP of *nce102Δ* cells from Fig. S1 in 3° angle increment (−30 to +30°) were calculated and joined. Presentation speed: 10 frames/s.
(AVI)
