# Supplementary figures and images for: Distribution of Cortical Endoplasmic Reticulum Determines Positioning of Endocytic Events in Yeast Plasma Membrane
Source: PLoS One. 2012 Apr 9;7(4):e35132. doi: 10.1371/journal.pone.0035132 (PMC3322162; doi:10.1371/journal.pone.0035132)

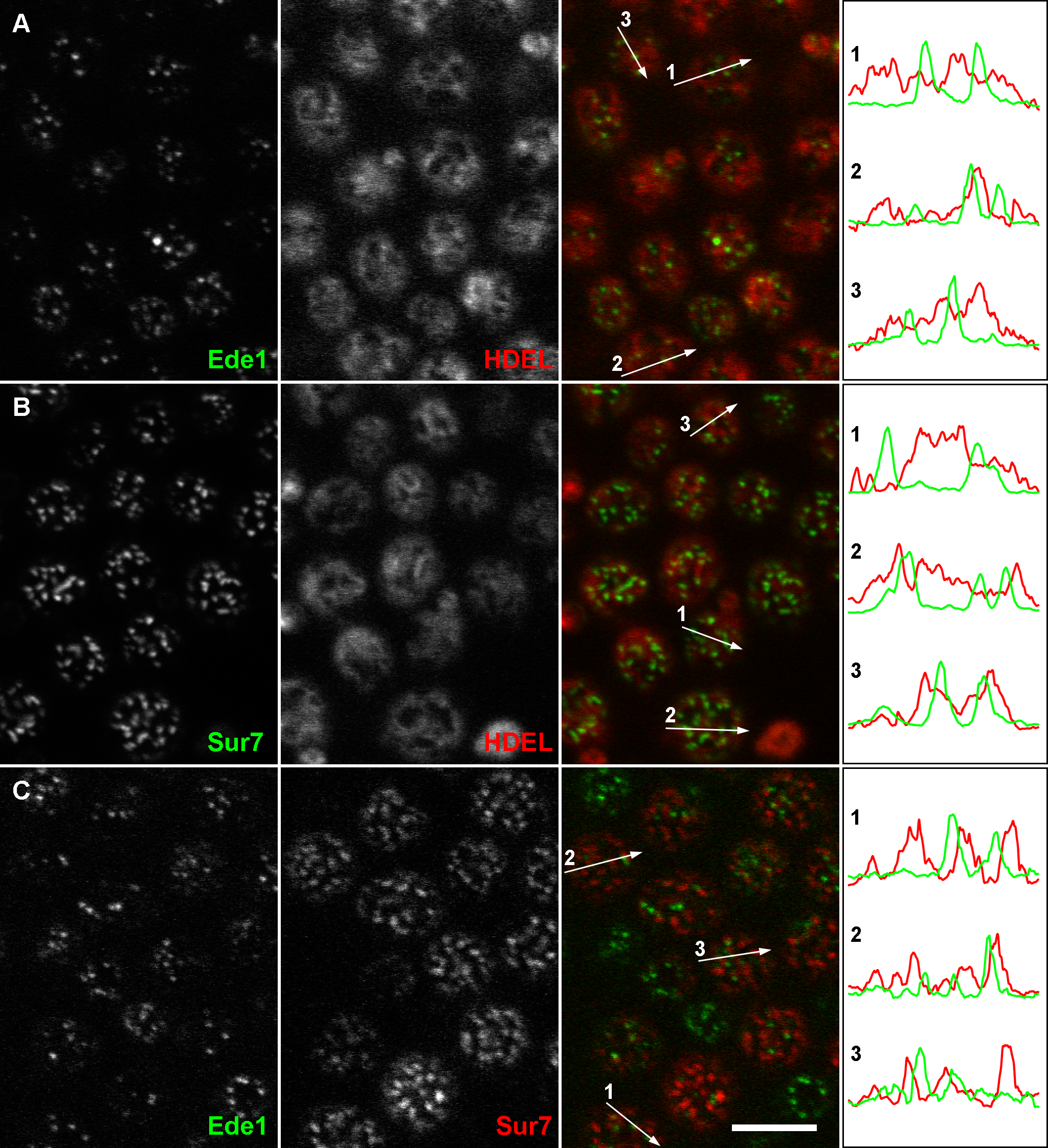

Supplement: Figure S1 — Endocytosis is initiated in the ER free zones of the plasma membrane in W303-1A cells. Mutual localization of Ede1-GFP, a marker of early stages of endocytosis, and cortical ER visualized by ss-dsRed-HDEL was performed. Only rare colocalization events were detected (A). Similarly, cortical ER network and initiation sites of endocytosis were not colocalized with MCC domains marked with Sur7-GFP (B) and Sur7-mRFP (C), respectively. Tangential confocal sections of W303-1A cells expressing fluorescently labeled proteins are presented. Fluorescence intensity profiles along the numbered arrows were scaled to the same range in the red and green channels. Bar: 5 µm. (TIF) [file pone.0035132.s001.tif]

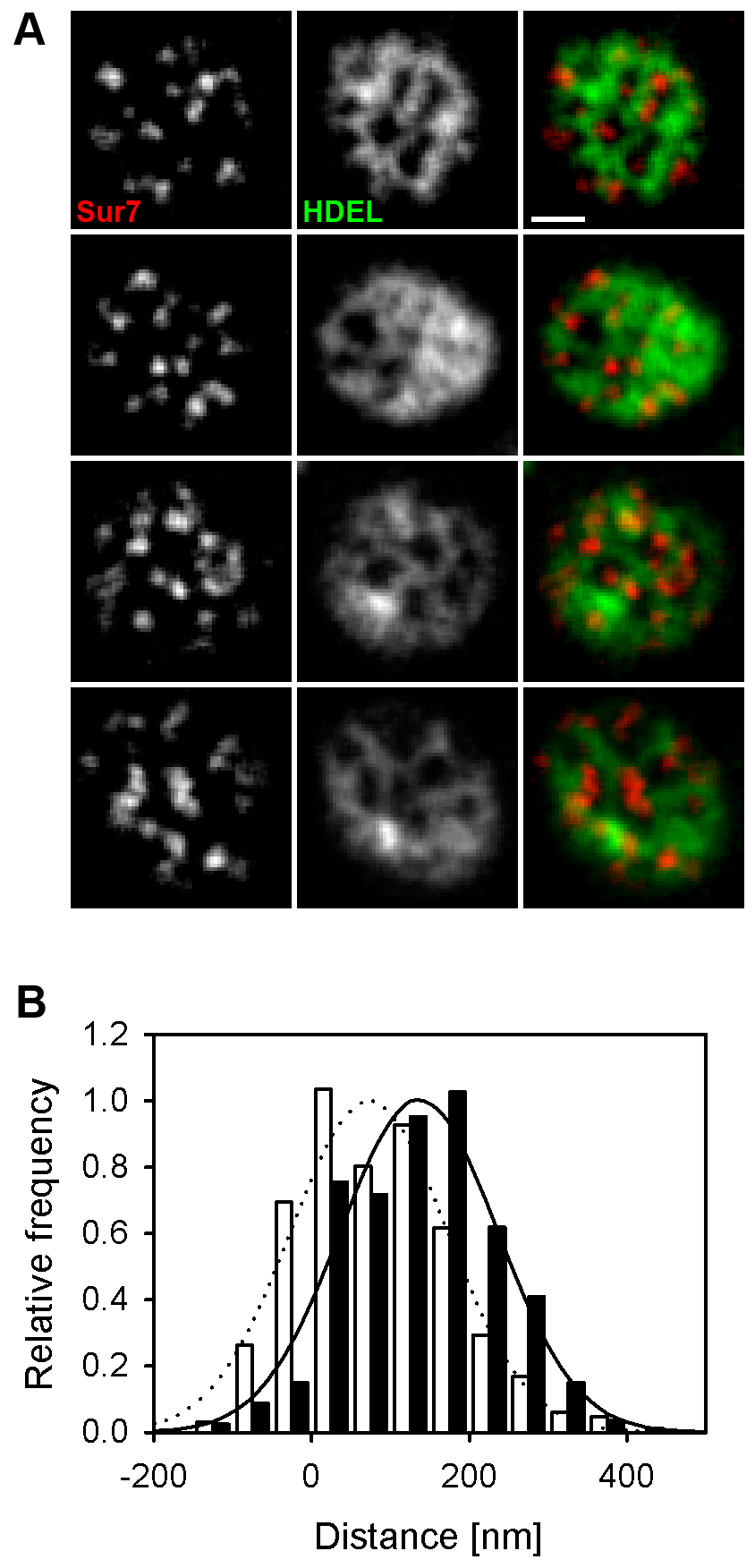

Supplement: Figure S2 — Distribution of MCC domains through the holes in the cortical ER pattern. In tangential confocal sections of individual cells expressing Sur7-mCherry and GFP-HDEL (A), the minimal distance of the Sur7 labeled MCC domains from the cortical ER boundary was measured. The histogram of the measured distances (full bars in B; 399 foci in 64 cells were analyzed) was compared to the distribution of the distances of model foci randomly positioned in the plasma membrane (empty bars in B; 320 foci in 100 cells; see Methods for details). The Gaussian fits of the distributions are also depicted (Sur7 solid, randomly positioned foci dotted). In order to maximize the accuracy of the distance measurements, for all the measurements we chose only the foci located to easily discernible ER holes positioned in central parts of the tangential confocal sections, so that the entire borders of the holes could be traced Bar: 1 µm. (TIF) [file pone.0035132.s002.tif]

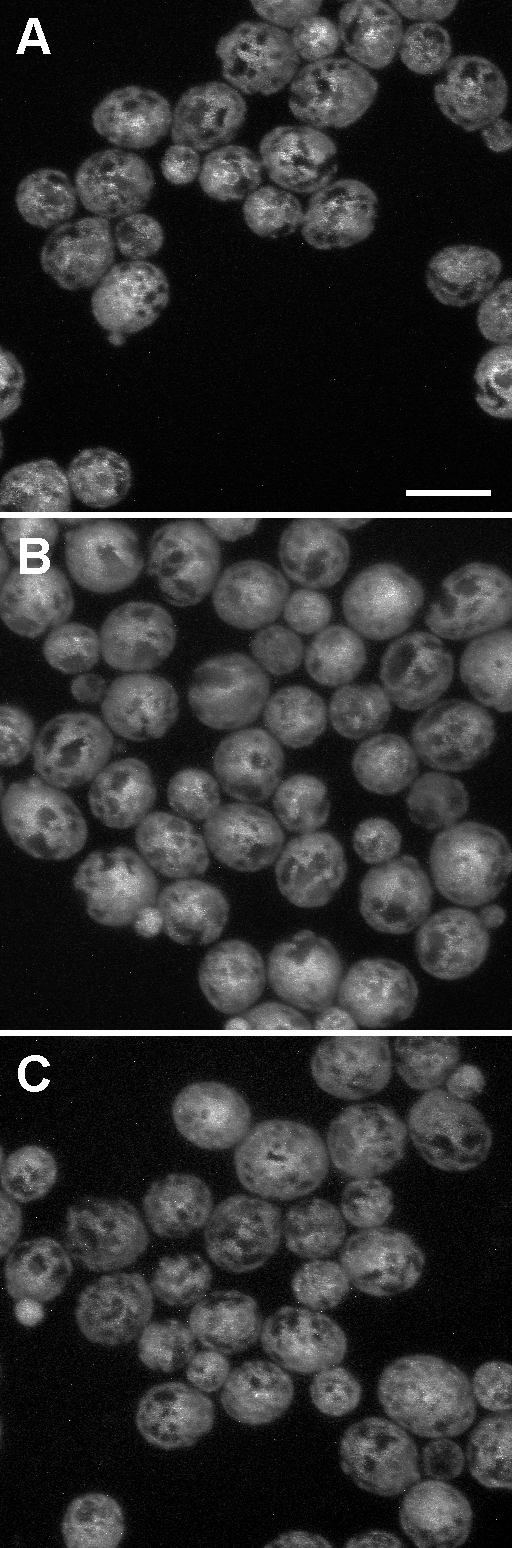

Supplement: Figure S3 — Cortical ER pattern in MCC-defective strains. Transparency projections (LSM Image Browser) of ER patterns in BY4741, pil1Δ and nce102Δ cells expressing ss-GFP-HDEL and Sur7-mCherry markers are compared. Only the green (ER) fluorescence pattern is presented. More projections of the same cells see also in Movies S1,S2,S3. Bar: 5 µm. (TIF) [file pone.0035132.s003.tif]

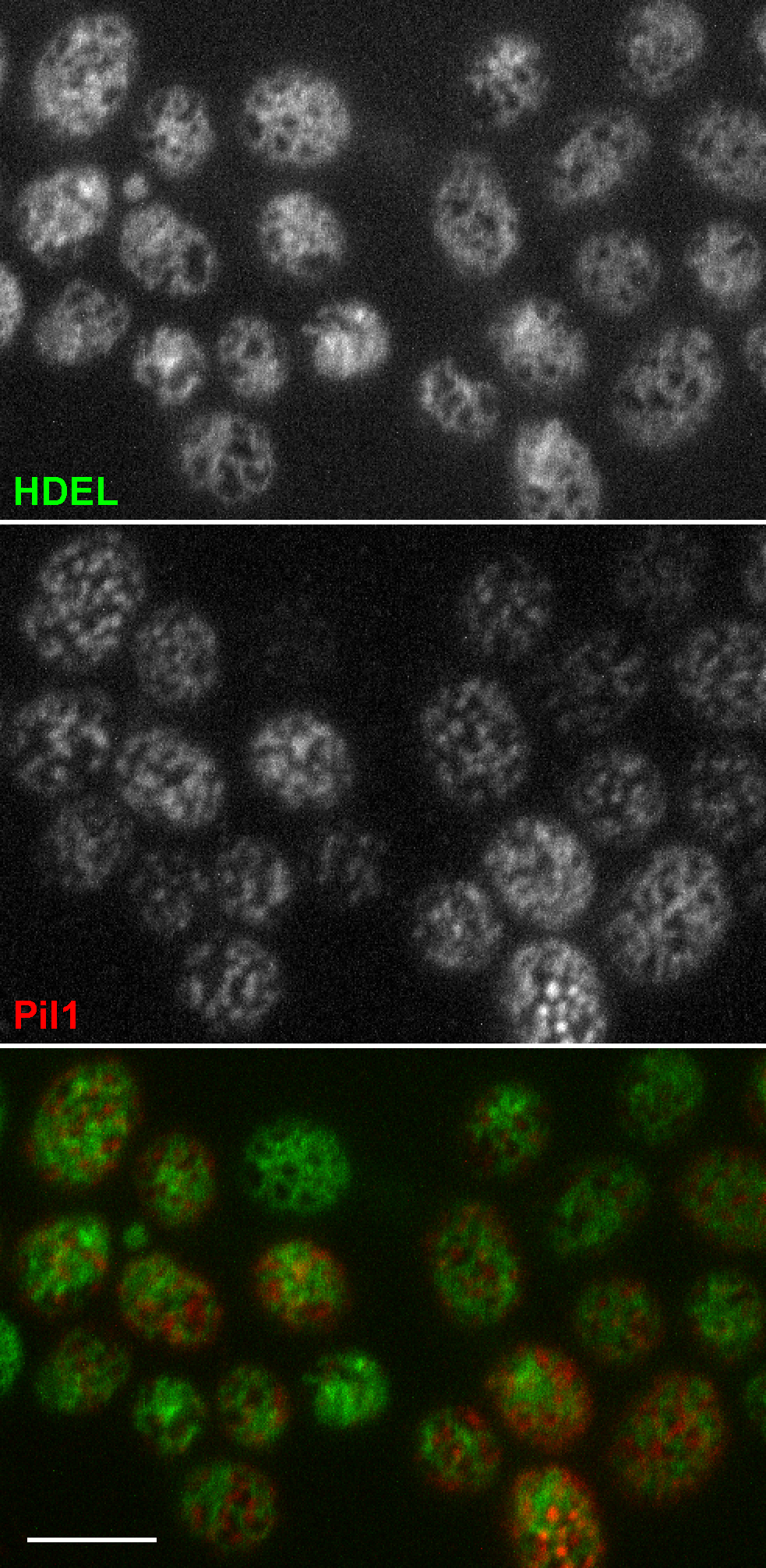

Supplement: Figure S4 — Overexpression of Pil1 leads to increased fragmentation of the cortical ER pattern. Cells co- expressing ss-GFP-HDEL (green) and Pil1-mRFP (red) under a strong promoter (strain VSY177) were observed. Compare the number of MCC/eisosomes and the number of cortical ER holes with those of wild type (Fig. 1) and pil1Δ cells (Fig. 5). Superposition of two consecutive confocal sections is presented. Bar: 5 µm. (TIF) [file pone.0035132.s004.tif]

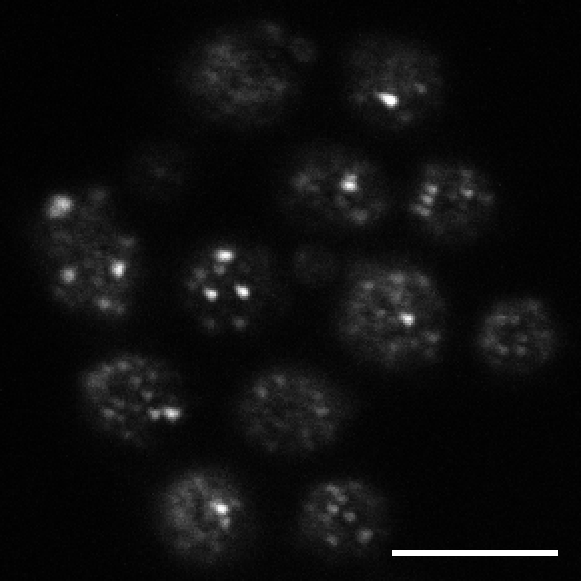

Supplement: Figure S5 — Distribution of Sur7 in pil1Δ cells. Tangential confocal sections of pil1Δ cells expressing ss-dsRed-HDEL and Sur7-GFP markers (only green fluorescence channel visible) are presented. Note that, in addition to large and intensive “eisosome remnants”, smaller and less intensive local accumulations of Sur7-GFP are discernible in the surrounding membrane. Bar: 5 µm. (TIF) [file pone.0035132.s005.tif]

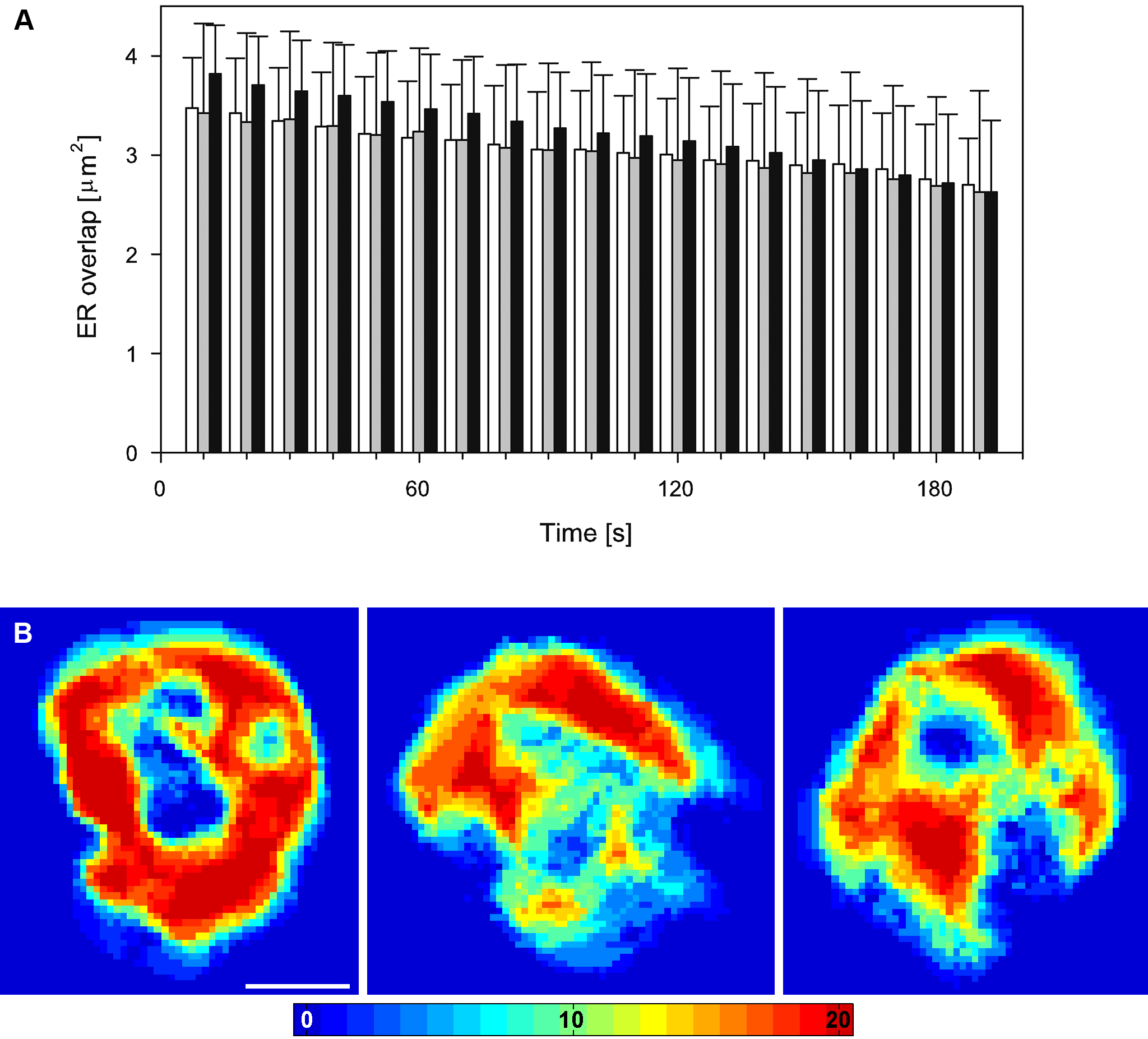

Supplement: Figure S6 — Speed of cortical ER movement is not affected in MCC defective strains. The speed of the cortical ER movement was measured as a decrease in the mutual overlap of the ss-GFP-HDEL patterns detected in living BY4741 (white), pil1Δ (grey) and nce102Δ (black) cells (n>30) after an increasing interval of time (A). The dynamics of cortical ER was followed in a time-lapse series of 20 tangential confocal sections of pil1Δ cells expressing ss-GFP-HDEL together with Sur7-mCherry (rate: 10 s/frame). For better lucidity, the red fluorescence channel (MCC/Sur7-mCherry) is not shown. The data were processed and binarized as shown in Fig. 3 and all twenty binarized frames were superimposed to visualize the local dynamics of cortical ER. Three out of 30 cells analyzed are presented in false colors denoting the number of frames in the series in which cortical ER was detected (B). Bar: 1 µm. (TIF) [file pone.0035132.s006.tif]
