# Supplementary material for: Distribution of Cortical Endoplasmic Reticulum Determines Positioning of Endocytic Events in Yeast Plasma Membrane
Source: PLoS One. 2012 Apr 9;7(4):e35132. doi: 10.1371/journal.pone.0035132 (PMC3322162; doi:10.1371/journal.pone.0035132)
Supplement: Table S1 — Strains used in this study. (DOC) [file pone.0035132.s007.doc]

**Table S1. Strains used in this study.**

| **Strain** | **Genotype** | **Source** |
| --- | --- | --- |
| BY4741 | *MAT*a *his3Δ1 leu2Δ0 met15Δ0 ura3Δ0* | Brachmann et al, 1998^*^ |
| W303-1A | *MATa leu2-3,112 trp1-1 can1-100 ura3-1 ade2-1 his3-11,15* | Thomas and Rothstein, 1989^**^ |
| GYS91 | BY4741 *nce102::kanMX4* | EUROSCARF |
| GYS130 | BY4741 *pil1::kanMX4* | EUROSCARF |
| VSY 182 | W 303-1A Ede1::GFP::URA3 (YIp211); ss-dsRed-HDEL::TRP1 (YIp204) | This study |
| VSY 24 | W303-1A Sur7::GFP::URA3 (YIp211); ss-dsRed-HDEL::TRP1(YIp204) | This study |
| VSY 40 | W303-1A Sur7::mRFP::LEU2 (YIp128); Ede1::GFP::URA3 (YIp211) | This study |
| VSY 26 | BY4741 Ede1::GFP::URA3 (YIp211); Sur7::mRFP::LEU2 (YIp128) | This study |
| VSY103 | BY4741 Sur7::mCherry::URA3 (YIp211); TRP1::ss-GFP-HDEL::LEU2 (YIp128) | This study |
| VSY99 | BY4741 Ede1::GFP::URA3 (YIp211); TRP1::ss-dsRed-HDEL::LEU2 (YIp128) | This study |
| VSY98 | BY4741 Sur7::GFP::URA3 (YIp211); TRP1::ss-dsRed-HDEL::LEU2 (YIp128) | This study |
| VSY 107 | GYS91 Sur7::mCherry::URA3 (YIp211); TRP1::ss-GFP-HDEL::LEU2 (YIp128) | This study |
| VSY 83 | GYS91 Sur7::GFP::URA3 (YIp211); TRP1::ss-dsRed-HDEL::LEU2 (YIp128) | This study |
| VSY 134 | GYS91 Ede1::GFP::URA3 (YIp211); TRP1::ss-dsRed-HDEL::LEU2 (YIp128) | This study |
| VSY 108 | GYS130 Sur7::mCherry::URA3 (YIp211); TRP1::ss-GFP-HDEL::LEU2 (YIp128) | This study |
| VSY 85 | GYS130 Sur7::GFP::URA3 (YIp211); TRP1::ss-dsRed-HDEL::LEU2 (YIp128) | This study |
| VSY 135 | GYS130 Ede1::GFP::URA3 (YIp211); TRP1::ss-dsRed-HDEL::LEU2 (YIp128) | This study |
| VSY 177 | BY4741 TRP1::ss-GFP-HDEL::LEU2 (YIp128); pVTU100-Pil1-mRFP | This study |

^*^ Brachmann et al. (1998), Yeast 14:115-32

^**^ Thomas & Rothstein (1989), Cell 56:619-630
